# Rising Catastrophic Expenditure on Households Due to Tuberculosis: Is India Moving Away From the END-TB Goal?

**DOI:** 10.3389/fpubh.2021.614466

**Published:** 2021-02-15

**Authors:** Banuru Muralidhara Prasad, Jaya Prasad Tripathy, V. R. Muraleedharan, Jamhoih Tonsing

**Affiliations:** ^1^International Union Against Tuberculosis and Lung Disease, The Union South East Asia Office, New Delhi, India; ^2^Department of Community Medicine, All India Institute of Medical Sciences, Nagpur, India; ^3^Humanities and Social Science Department, Indian Institute of Technology Madras, Chennai, India

**Keywords:** catastrophic expenditure, household, tuberculosis, national sample survey, India

## Abstract

**Introduction:** One of the targets of the END-TB strategy is to ensure zero catastrophic expenditure on households due to TB. The information about household catastrophic expenditure is limited in India and, therefore difficult to monitor. The objective is to estimate household and catastrophic expenditure for Tuberculosis using national sample survey data.

**Methods:** For arriving at out-of-pocket expenditure due to tuberculosis and its impact on households the study analyzed four rounds of National Sample Survey data (52nd round-1995–1996, 60th round-2004–2005, 71st round-2014–15, and 75th round 2017–2018). The household interview survey data had a recall period of 365 days for inpatient/ hospitalization and 15 days for out-patient care expenditure. Expenditure amounting to >20% of annual household consumption expenditure was termed as catastrophic.

**Results:** A 5-fold increase in median outpatient care cost in 75th round is observed compared to previous rounds and increase has been maximum while accessing public sector. The overall expense ratio of public v/s private is 1:3, 1:4, 1:5, and 1:5, respectively across four rounds for hospitalization. The prevalence of catastrophic expenditure due to hospitalization increased from 16.5% (52nd round) to 43% (71st round), followed by a decline to 18% in the recent 75th round.

**Conclusion:** Despite free diagnostic and treatment services offered under the national program, households are exposed to catastrophic financial expenditure due to tuberculosis. We strongly advocate for risk protection mechanisms such as cash transfer or health insurance schemes targeting the patients of tuberculosis, especially among the poor.

## Key Points

The nationally representative survey sample data from 1995–96 to 2017–18 was analyzed to estimate household and catastrophic expenditure for TB. The expenditure ratio public v/s private was about 5-folds for outpatient care and similar trend was observed for hospitalization. Poor household face more catastrophic expenditure while hospitalization services are accessed in private sector and there is a need for risk protection mechanisms.

## Introduction

Over the decades, Tuberculosis (TB) disease continues to cause suffering and is still one of the leading causes of death in the world. In 2019 alone, more than 10 million people had suffered due to TB, which has claimed nearly 1.2 million lives (1). Given this scenario, the global community has pledged to End-TB by 2035 with one of the main targets is to ensure households face “zero” catastrophic costs due to TB (2).

Systematic reviews across low- and middle-income countries showed mean total costs of TB ranging from $1 to 8,198, with >60% accounting for indirect cost (3–5). There is a need to quantify and understand the financial burden on TB patients in-order to adopt appropriate policies to realize the global target of having zero households incurring catastrophic costs because of TB disease.

India has nearly one-quarter of the global TB burden. Despite free TB diagnosis and treatment in the public sector, TB morbidity and mortality pose an enormous economic burden to patients, household, and society. Limited evidence shows that each year, a TB patient loses on average 3–4 months of work and up to 30% of household earnings (6–9). These studies were limited to a specific geographical area or a particularly vulnerable group of patients. The studies mainly included patients who were enrolled in the National TB Program for treatment. However, patients availing diagnostic and treatment services from private sector and those on non-DOTS regimens are often missed, thus limiting the representativeness of the estimates to the entire population of patients with TB.

In a country like India, where government spending on health is low and with a larger presence of private providers; patients often spend out-of-pocket (OOP) to meet the health care needs. In this scenario, we have limited estimates of expenditure due to TB, both in the public and in the private sector. The data of nationally representative surveys conducted by the National Sample Survey Office (NSSO), provides us an opportunity to understand household expenditure patterns. As the NSSO conducts periodic surveys, it also allows us to compare out-of-pocket expenditure for TB care across different periods.

The objective of the current study is to estimate household and catastrophic expenditure for Tuberculosis using national sample survey data. In addition, the study also estimates the expenditure for out-patient care and hospitalization due to TB and its catastrophic impact on households in both the public and private sectors, compared across periods.

## Methods

### Study Design

This is a secondary data analysis of four rounds of surveys conducted by the NSSO from 1995–1996 to 2017–2018 – (a) Survey on Health Care: NSS 52nd Round, 1995–1996 (b) Survey on Morbidity and Health Care: NSS 60th Round, January 2004 - June 2005, (c) Social Consumption - Health Survey: NSS 71st Round, January - June 2014, (d) Social Consumption in India – Health: NSS 75th Round, July 2017-June 2018. The periodic surveys with similar methodology for health is commissioned by Ministry of Statistics and Program Implementation, Government of India.^1^

### Data Source

The NSSO surveys collect data on morbidity profile of the population, utilization of public and private health care facilities, and expenditure incurred on various ailments requiring hospitalized and non-hospitalized care. The surveys collected household details, socio-demographic particulars, and details of ailments and medical treatment received. Though the methodology used across surveys are similar, there are few variations in the design and sampling were present across the four rounds, these variations are focused on improving the data quality and do not affect our analysis of the data with regards to TB.

Out of Pocket Expenditure (OOPE) for each episode of hospitalization (as inpatient care –in-patient department- IPD) and outpatient or day-care was recorded. Detailed expenditure was available for both direct and indirect costs. Direct costs included expenditure related to drugs (allopathic and other systems of medicine), diagnostic tests (including ECG, X-ray, and pathological tests); professional fees for doctors; payments to hospital/institution; other medical expenses (physiotherapy, personal medical appliances, blood, oxygen, attendant charges, etc.). Indirect costs included transport for patients and other accompanying persons, food-related expenses, lodging charges, and others.

The data from respondents were collected for hospitalization and outpatient. The recall period was 365 days for assessing inpatient hospitalization expenditure, 15 days for outpatient expenditure, and one month for household consumption expenditure. OOP expenditure for hospitalization and outpatient care was considered separately. The OOP expenditure, either due to outpatient care or hospitalization amounting to more than 20% of annual household consumption expenditure was termed as “catastrophic” (10). Catastrophic expenditure assessed thus was for each episode of outpatient care or hospitalization. Ascertainment of TB in the survey was based on self-report by the respondent. All those who reported hospitalization or having availed outpatient care due to TB within the said reference period as mentioned above were included in this study.

### Data Analysis

Data were imported into STATA version 12.0 for analysis (STATA IC/12.0 for Windows, StataCorp LP, USA). The master data sheets were then analyzed using SPSS 16.0 (SPSS Inc. Released 2007. SPSS for Windows, Version 16.0. Chicago, SPSS Inc.). Each episode of hospitalization or outpatient visit due to TB was the unit of analysis. The study population was divided into five quintiles based on monthly per capita consumption expenditure (MPCE). Median (interquartile range) expenditure and the median proportion of catastrophic expenditure across four rounds of the survey are presented for each of the five MPCE quintiles and type of health facility (public and private). The cost of outpatient care and hospitalization has been given separately. Due to the complex multistage sampling design, estimates were derived by applying sampling weights provided in the data sets.

The expenditure estimates of the 52nd round, 60th round, and 71st round have been equated to the estimates of the 75th round using the Consumer Price Index (CPI) published by the World Bank for India (11). Accordingly, the conversation rate values for the 52nd round, 60th round, and 71st were 4.23, 2.52, and 1.139 respectively. The values in Indian rupees are converted to US dollars (USD) at the annual average rate of 65.12 for the reference year 2017 as published by The World Bank (12).

### Ethics Approval

The Ethics Advisory Group of the International Union Against Tuberculosis and Lung Disease (The Union), Paris, France, determined that ethics clearance was not required for this study as it involved analysis of secondary data available in the public domain.

## Results

### Cost of Outpatient Care

A total of 580, 671, 299, and 275 members of household in the 52nd, 60th, 71st, and 75th rounds, respectively, who accessed outpatient care in the last 15 days due to TB were included in this analysis. The outpatient care proportion in public sector increased from 46, 59, 55, and 61% in respective rounds.

In the recent rounds 71st & 75th round, the overall median cost incurred per outpatient care was USD 4 (3–15) & USD 22 (7–61) a five-time increase in cost incurred. In the public sector, each episode of outpatient care cost US$ 12 (1–141) as per the latest 75th round and US$ 2 (1–4) in the 71st round. Outpatient care costed more than twice in the private sector in the 75th round [US$ 22 (16–31)] compared to the 71st round [US$ 9 (4–21)]. The cost of outpatient care has seen a downward trend when compared to previous rounds of surveys in both the public and private sectors except for the recent 75th round (Table 1).

**Table 1:** Comparison of cost of each episode of outpatient care due to tuberculosis across wealth quintiles in four national surveys of India from 1995–1996 to 2017–2018.

	**Overall expenditure**				**Public sector**				**Private sector**			
**Quintile**	**52nd round**	**60th round**	**71st round**	**75th round**	**52nd round**	**60th round**	**71st round**	**75th round**	**52nd round**	**60th round**	**71st round**	**75th round**
	***N* = 375**	***N* = 127**	***N* = 287**	***N* = 275**	***N* = 172**	***N* = 75**	***N* = 159**	***N* = 168**	***N* = 180**	***N* = 52**	***N* = 128**	***N* = 89**
First quintile	2 (0–17)	6 (1–17)	3 (3–5)	16 (10–61)	3 (2–6)	3 (1–14)	3 (1–3)	2 (2–17)	20 (7–42)	6 (3–19)	4 (3–10)	16 (16–61)
Second quintile	10 (0–17)	6 (2–9)	9 (4–21)	26 (22–141)	10 (4–20)	2 (2–5)	3 (1–10)	141 (141–141)	14 (14–17)	9 (6–14)	11 (4–21)	22 (22–26)
Third quintile	1 (0–16)	13 (3–21)	16 (6–16)	8 (2–9)	1 (1–5)	1 (1–9)	6 (1–54)	8 (4–8)	20 (12–59)	13 (12–22)	16 (7–16)	6 (2–9)
Fourth quintile	7 (0–15)	62 (37–66)	4 (1–25)	1 (1–10)	15 (7–23)	74 (6–74)	1 (1–4)	1 (1–1)	13 (9–23)	62 (62–62)	25 (16–25)	10 (10–30)
Fifth quintile	13 (3–33)	44 (15–58)	1 (1–4)	48 (8–372)	8 (3–12)	44 (15–206)	1 (1–1)	0 (0–3)	49 (16–130)	46 (19–46)	14 (9–27)	372 (35–372)
Overall	7 (0–20)	12 (3–37)	4 (3–15)	22 (7–61)	7 (3–14)	3 (1–15)	2 (1–4)	12 (1–141)	16 (13–42)	14 (8–62)	9 (4–21)	22 (16–31)

*52nd Round was conducted b/w 1995 and 96 and CPI unit used is 4.23 to equate to 2017 value. 60th Round was conducted b/w 2004 and CPI unit used is 2.52 to equate to 2017 value. 71st Round was conducted b/w 2014 and 2015 and CPI unit used is 1.139 to equate to 2017. The annual average of USD exchange rate for 2017 is 65.12 to INR is used across the rounds. The figures are in median expenditure and interquartile range (IQR) is in parentheses*.

From all four rounds, on an average 53% of the participants sought outpatient care in the public sector and 44% in the private sector. The outpatient services in the public sector were mostly accessed by the population from the 1st quintile, while the private sector was accessed mainly by the 5th quintile across all four rounds. The total overall medical expenditure due to outpatient visit has shown a drop in the 71st Round compared to the previous rounds. The overall expenditure ratio Public v/s Private is 1:3, 1:4, 1:5, and 1:5 across 52nd, 60th, 71st, and 75th round respectively.

Overall, 2.3% of households faced catastrophic expenditure toward outpatient care as a result of TB, 1.7% in the public sector, and 3.1% in the private sector as per the NSSO 71st round. In the recent 75th round, the prevalence of catastrophic expenditure was zero in both the public and private sectors. The prevalence of catastrophic expenditure has increased from 0.3% (52nd round) to 2.3% (71st round), dipping to zero in the 75th round (Table 2).

**Table 2:** Prevalence of catastrophic expenditure as a result of one episode of outpatient care due to tuberculosis across wealth quintiles in four national surveys of India from 1995–1996 to 2017–2018.

	**Overall expenditure**				**Public sector**				**Private sector**			
	**52nd round**	**60th round**	**71st round**	**75th round**	**52nd round**	**60th round**	**71st round**	**75th round**	**52nd round**	**60th round**	**71st round**	**75th round**
First quintile	0	2.6	4.5	0	0	0	5.4	0	0	10	3.8	0
Second quintile	0	0	3.8	0	0	0	0	0	0	0	6.9	0
Third quintile	0.8	0	0	0	0	0	0	0	3	0	0	0
Fourth quintile	0.8	0	0	0	0	0	0	0	2	0	0	0
Fifth quintile	0	0	0	0	0	0	0	0	0	0	0	0
Overall	0.3	1	2.3	0	0	0	1.7	0	1	2.7	3.1	0

### Cost of Hospitalization

A total of 756 (500 public, 241 private), 901 (550 public, 352 private), 610 (310 public, 210 private), and 610 (412 public, 198 private)participants from the 52nd, 60th, 71st, and 75th round, respectively, who were hospitalized in the last 365 days due to TB were included in this analysis. A total of 66%, 61%, and 60% of the participants sought hospitalization in the public sector in the 52nd, 60th, and the 71st round, respectively. In the 75th round, the hospitalization increased to 67% in the public sector; population from 1st quintile accessed more of the public sector and 5th quintile accessed the private sector for inpatient care across four rounds (Supplementary Table 1).

In the 52nd round & 75th round, the median total cost as a result of hospitalization due to TB has remained the same at USD 111(40–286) toUSD110 (34–279). The median cost of hospitalization in the public has shown a decline from USD 79 (27–172), 87 (32–221), 44(20–149), and a slight increase in the 75th round to USD 56 (18–140). The hospitalization expenses in the private sector have increased from US$ 231 in the 52nd round to US$ 261 in the 75th round (Table 3). Medicines form the major proportion of medical care costs of hospitalization. The expenditure on medicines was 31% of total hospitalization cost in the 71st round, and this declined to 29% in the 75th round. A gross reduction in expenditure on medicines was seen in the 1st quintile, while the services were availed in the public sector (41% in 71st round to 26% in 75th round). Given this, the overall expenditure ratio – Public v/s Private is at 1:3, 1:4, and 1:6 times across 52nd, 60th, and 71st rounds, respectively.

**Table 3:** Comparison of cost of each episode of hospitalization due to tuberculosis across wealth quintiles in four national surveys of India from 1995–1996 to 2017–2018.

	**Overall expenditure**				**Public sector**				**Private sector**			
	**52nd round**	**60th round**	**71st round**	**75th round**	**52nd round**	**60th round**	**71st round**	**75th round**	**52nd round**	**60th round**	**71st round**	**75th round**
	***N* = 755**	***N* = 901**	***N* = 520**	***N* = 610**	***N* = 500**	***N* = 550**	***N* = 310**	***N* = 610**	***N* = 241**	***N* = 351**	***N* = 210**	***N* = 610**
First quintile	66 (26–133)	103 (45–285)	72 (35–171)	76 (34–346)	60 (20–130)	69 (21–199)	54 (15–135)	65 (15–172)	81 (36–184)	202 (82–359)	175 (84–733)	281 (110–748)
Second quintile	98 (41–218)	165 (66–310)	84 (28–194)	88 (27–249)	68 (23–105)	94 (46–172)	28 (28–101)	34 (4–146)	286 (107–481)	310 (198–600)	192 (84–297)	249 (161–825)
Third quintile	144 (33–312)	116 (35–275)	91 (46–264)	169 (48–304)	73 (7–260)	62 (12–213)	70 (25–199)	65 (28–120)	237 (144–377)	231 (116–349)	161 (98–358)	261 (261–396)
Fourth quintile	136 (62–293)	331 (97–721)	116 (15–337)	100 (33–217)	130 (62–218)	96 (43–395)	17 (6–88)	58 (28–150)	137 (34–345)	581 (314–2,014)	252 (126–675)	260 (130–439)
Fifth quintile	375 (183–985)	236 (106–910)	332 (65–350)	110 (43–338)	504 (189–985)	181 (50–910)	334 (21–350)	49 (18–134)	293 (183–1,014)	271 (153–910)	332 (105–341)	244 (110–619)
Overall	111 (40–286)	170 (60–411)	91 (28–285)	110 (34–279)	79 (27–172)	87 (32–221)	44 (20–149)	56 (18–140)	231 (81–390)	310 (143–620)	233 (93–364)	261 (163–484)
Equity ratio	5.7	2.3	4.6	1.4	8.4	2.6	6.2	−0.8	3.6	1.3	1.9	−0.9

*52nd Round was conducted b/w 1995 and 1996 and CPI unit used is 4.23 to equate to 2017 value. 60th Round was conducted b/w 2004 and 2005 and CPI unit used is 2.52 to equate to 2017 value. 71st Round was conducted b/w 2014 and 2015 and CPI unit used is 1.139 to equate to 2017. The annual average of USD exchange rate for 2017 is 65.12 to INR is used across the rounds. The figures are in median expenditure and interquartile range (IQR) is in parentheses*.

Overall, 43% of the households faced catastrophic expenditure due to hospitalization as a result of TB in the 71st round, 32% in the public sector, and 62% in the private sector, whereas it declined to 18% in the 75th round. The prevalence of catastrophic expenditure has been increasingacross the three surveys from 16.5% in the 52nd round to 43% in the 71st round. A similar increase has been observed both in the public and the private sector (Table 4).

**Table 4:** Prevalence of catastrophic expenditure as a result of one episode of hospitalization due to tuberculosis across wealth quintiles in four national surveys of India from 1995–1996 to 2017–2018.

	**Overall expenditure**				**Public sector**				**Private sector**			
	**52nd round**	**60th round**	**71st round**	**75th round**	**52nd round**	**60th round**	**71st round**	**75th round**	**52nd round**	**60th round**	**71st round**	**75th round**
First quintile	6.2	33.7	56	25	5	27	46	16	11	44	81	46
Second quintile	14.8	33	43	20	8	24	33	10	35	49	56	30
Third quintile	18.5	40.1	32	26	13	29	22	7	28	55	47	49
Fourth quintile	17.6	32.6	34	13	15	21	15	4.5	21	52	62	24
Fifth quintile	36.6	21.2	32	20	38	20	20	6	36	22	46	33
Overall	16.5	33.5	43	18	12	25	32	9	26	46	62	36

## Discussion

This study estimates the cost of each episode of outpatient care and hospitalization due to TB in both the public and private sectors and its catastrophic impact on households. The study also compares the cost estimates across four surveys conducted in different periods. The main findings of the study are (a) there is an increase in household expenditure especially when the services are accessed in private sector (b) catastrophic expenditure is evident among the poor households while accessing hospitalization services.

First, every two out of five episodes of hospitalization due to TB have a catastrophic impact on households according to the 71st round of survey in 2014. This has declined to 18% as per the latest 75th round in 2017–18. A similar finding has also been observed in the case of expenditure due to outpatient care. This is encouraging and could be attributed to the various scheme and health system strengthening under the National TB Elimination Program (NTEP), although further studies are required to confirm this. Previous studies in India, China, Peru, and few African countries have also reported significant cost of care and impact on households due to TB care (3, 7–9, 13–17). A recent cross-sectional study of 455 individuals with TB in South India showed that despite the implementation of free diagnostic and treatment services under a national TB control program, one-third of TB-affected households still experienced catastrophic costs (18). A similar cross-sectional study among 450 TB patients in New Delhi, India reported that 7% of patients registered under the national TB program experienced catastrophic expenditure, with a large proportion being accounted by indirect costs (19). Poornima et al. also reported significant cost of care due to TB in programmatic settings with one in eight patients experiencing catastrophic expenditure (17). A direct comparison of the figures in these studies would not be fair as they involved different cut-offs of catastrophic expenditure, varying periods of follow-up and different methodologies. Nevertheless, it is unequivocal that despite free diagnosis and treatment offered for TB in the public sector, patients with TB incur huge healthcare costs.

Second, despite the various initiatives taken by the Government of India in the last few decades toward TB control, the cost of care, and its financial impact on their households has been increasing. This is alarming, especially at a time when we have set ourselves a target of zero catastrophic expenditure. A recent study from south India, highlighted that, 31% of households face catastrophic cost even when the services are provided free of cost (20).

There were a few strengths in this study. First, this was a population-based study including TB patients from both public and the private sector, thereby lending representativeness of the estimates to the entire population of patients with TB. Second, a standard robust methodology was adopted in all the four surveys also allows valid comparison. Third, a comparison of estimates from four different surveys allows analysis of trends over the last three decades.

There were some limitations as well. First, the cost estimates presented in this study did not capture expenditure during the entire duration of TB treatment which is a minimum of 6 months. This might have underestimated the study results. Second, over-reporting of expenditure is common which could have led to overestimating the proportion of households experiencing catastrophic health costs. However, over-reporting of household consumption expenditure, which is the denominator for estimating catastrophic expenditure, is also likely. This could cause underestimation of the catastrophic impact, thereby nullifying the above effect. Third, there is no information on the clinical profile of TB patients such as type of TB, drug resistance pattern, other co-morbidities, and disease severity which would have confounded the results. Fourth, data on household income loss (loss of income of patient and caregivers) could not be assessed as it was not captured in the survey data. Fifth, the reasons for high OOP expenditure and catastrophic expenditure could not be explored as it was the beyond the scope of this study.

Despite these limitations, the study findings have important policy implications. First, financial support in terms of cash transfer or health insurance is needed to offset the impact of expenditure due to TB. With the Direct Benefit Transfer (DBT) Scheme and the Ayushman Bharat insurance scheme, it seems that things are moving in the right direction. However, targeting the needy and the poor is essential under these schemes. Previous assessments in India have highlighted several challenges to the implementation of the DBT scheme which needs to be addressed to improve uptake and efficiency (21, 22). Also, considering the burden of expenditure due to TB, the amount transferred under DBT would not be enough to provide sufficient compensation for most patients as echoed in other studies (18).

Second, the median OOP expenditure in the private sector was nearly 5 times higher than the public sector. This is due to the burgeoning growth of the private sector in the health care market in the last few decades. The predominant role of the private sector in TB care is also echoed by previous studies. The private sector manages more than 50% of all TB cases, which cannot be ignored (18). Thus, the public sector needs to work together with the private players to provide TB care which is affordable and accessible to all. The public-private partnership initiatives under the TB program is a step in the right direction, although, it needs to be scaled-up using the recent guidance document (23).

## Conclusion

The study shows that a household with a member suffering from TB is exposed to significant financial risks which lead to catastrophic household expenditure, with a worsening trend over the last few decades. This is despite free diagnostic and treatment services offered under the national TB program. We strongly advocate for risk protection mechanisms targeting the poor; such as cash transfer or health insurance schemes. Future research is required to explore the reasons for high OOP expenditure due to TB in different settings, both quantitatively and qualitatively. We also need to understand the uptake and impact of various social protection schemes that are aimed at protecting catastrophic expenditure and correlate to subsequent rounds of National Sample Surveys.

## Data Availability Statement

Publicly available datasets were analyzed in this study. This data can be found here: http://www.icssrdataservice.in/Health_care.php.

## Author Contributions

BP, JPT, and VM conceptualized the paper and interpreted the data and critically reviewed the manuscript. BP and JPT analyzed and interpreted the data, drafted, reviewed, and edited the manuscript and took lead in drafting the manuscript. JT reviewed and provided key inputs to the manuscript. All the authors have read and approved the final version of the manuscript.

## Conflict of Interest

The authors declare that the research was conducted in the absence of any commercial or financial relationships that could be construed as a potential conflict of interest.

## Footnotes

^1^The Ministry of Statistics and Programme Implementation of Government of India, through National Sample Survey Office which was established as a survey organization in 1950 to conduct periodic surveys on various aspects around social, economic development, employment etc. The survey sampling framework is uniform across the survey -rounds. The 52nd Round titled as “Survey on Health Care: NSS 52nd Round, 1995–1996”adopted a two stage sampling design and collected data from a total of 120,942 households (71,284 in rural areas and 49,658 in urban areas). The subsequent surveys 60th Round titled as “Survey on Morbidity and Health Care,” 71st Round titled as “Social Consumption - Health Survey,” and 75th Round titled as “Social Consumption in India – Health” conducted stratified multistage sampling method. The methodology is detailed in the reports “Note on Sample Design and Estimation Procedure of NSS 75th Round.” In 60th round a total of 73,868 households were covered (in rural 47,302 and in urban 26,566) and in 71st round 65,932 households were covered with 36,480 from rural and 29,452 from urban areas. In the recent 75th round, the data was collected from 113,823 sample of households with 64,552 from rural areas and 49,271 from urban areas. The household interviewquestionnaires included 58 self-reported morbidity in 52nd round, 42 in the 60th round, and 61 in both 71st and 75th round.

## References

[1] World Health Organization. Global Tuberculosis Report 2018. Geneva: World Health Organization (2018).

[2] World Health Organization. The End TB Strategy: Global Strategy and Targets for Tuberculosis Prevention, Care and Control After 2015. Geneva: World Health Organization (2014).

[3] Ukwaja KN Modebe O Igwenyi C Alobu I. The economic burden of tuberculosis care for patients and households in Africa: a systematic review. Int J Tuberc Lung Dis. (2012) 16:733–9. 10.5588/ijtld.11.0193 22410546

[4] Barter DM Agboola SO Murray MB Bärnighausen T. Tuberculosis and poverty: the contribution of patient costs in sub-Saharan Africa – a systematic review. BMC Public Health. (2012) 12:980. 10.1186/1471-2458-12-980 23150901 PMC3570447

[5] Tanimura T Jaramillo E Weil D Raviglione M Lonnroth K. Financial burden for tuberculosis patients in low- and middle-income countries: a systematic review. Eur Respir J. (2014) 43:1763–75. 10.1183/09031936.00193413 24525439 PMC4040181

[6] Laxminarayan R Klein E Dye C Floyd K Darley S Adeyi O. Economic Benefit of Tuberculosis Control. Washington, DC: World Bank (2007).

[7] Muniyandi M Rao V Rao V Bhat J Yadav R Sharma R. Household catastrophic health expenditure due to tuberculosis: analysis from particularly vulnerable Tribal Group, Central India. Med Mycol Open Access. (2016) 2:9. 10.21767/2471-8521.100009

[8] Ananthakrishnan R Muniyandi M Jeyaraj A Palani G Sathiyasekaran BWC. Expenditure pattern for tb treatment among patients registered in an Urban Government DOTS Program in Chennai City, South India. Tuberc Res Treat. (2012) 2012:747924. 10.1155/2012/747924 23213507 PMC3507048

[9] Prasanna T Jeyashree K Chinnakali P Bahurupi Y Vasudevan K Das M. Catastrophic costs of tuberculosis care: a mixed methods study from Puducherry, India. Glob Health Action. (2018) 11:1477493. 10.1080/16549716.2018.1477493 29902134 PMC6008578

[10] Tripathy J Jagnoor J Prasad B Ivers R. Cost of injury care in India: cross-sectional analysis of National Sample Survey 2014. Inj Prev. (2018) 24:116–22. 10.1136/injuryprev-2017-042318 28724552

[11] World Bank. World Development Indicators. (2020). Retrieved from: https://data.worldbank.org/indicator/FP.CPI.TOTL?locations=IN&view=chart (accessed January 10, 2020).

[12] World Bank. World Development Indicators. (2020). Retrieved from: https://data.worldbank.org/indicator/PA.NUS.FCRF?locations=IN (accessed January 20, 2020).

[13] Laokri S Dramaix-Wilmet M Kassa F Anagonou S Dujardin B. Assessing the economic burden of illness for tuberculosis patients in Benin: determinants and consequences of catastrophic health expenditures and inequities. Trop Med Int Health. (2014) 19:1249–58. 10.1111/tmi.12365 25040399

[14] Verguet S Riumallo-Herl C Gomez GB Menzies NA Houben RMGJ Sumner T. Catastrophic costs potentially averted by tuberculosis control in India and South Africa: a modelling study. Lancet Glob Heal. (2017) 5:e1123–32. 10.1016/S2214-109X(17)30341-8 29025634 PMC5640802

[15] Batte C Kirenga B Katamba A Baena IG. Catastrophic total costs due to tuberculosis among affected households in Uganda; prevalence, drivers and policy implications. Eur Respir J. (2019) 54 (Suppl. 63):PA2793. 10.1183/13993003.congress-2019.PA279332938411

[16] Lu L Jiang Q Hong J Jin X Gao Q Bang H. Catastrophic costs of tuberculosis care in a population with internal migrants in China. BMC Health Serv Res. (2020) 20:832. 10.1186/s12913-020-05686-5 32887605 PMC7602335

[17] Poornima MP Shruthi MN Chingale AL Veena V Nagaraja SB Madhukeshwar AK. Cost of tuberculosis care in programmatic settings from Karnataka, India: is it catastrophic for the patients? Tuberc Res Treat. (2020) 2020:3845694. 10.1155/2020/3845694 32455013 PMC7238341

[18] Muniyandi M Thomas BE Karikalan N. Association of tuberculosis with household catastrophic expenditure in South India. JAMA Netw Open. (2020) 3:e1920973. 10.1001/jamanetworkopen.2019.20973 32049293 PMC11845097

[19] Sarin R Vohra V Singla N Thomas B Krishnan R Muniyandi M. Identifying costs contributing to catastrophic expenditure among TB patients registered under RNTCP in Delhi metro city in India. Indian J Tuberc. (2019) 66:150–7. 10.1016/j.ijtb.2018.10.009 30797274

[20] Patel BH Jeyashree K Chinnakali P Vijayageetha M Mehta KG Modi B. Cash transfer scheme for people with tuberculosis treated by the National TB Programme in Western India: A mixed methods study. BMJ Open. (2019) 9:e033158. 10.1136/bmjopen-2019-033158 31888934 PMC6936995

[21] Nirgude AS Kumar AMV Collins T Naik PR Parmar M Tao L. ‘I am on treatment since 5 months but I have not received any money': coverage, delays and implementation challenges of ‘Direct Benefit Transfer' for tuberculosis patients–a mixed-methods study from South India. Glob Health Action. (2019) 12:1633725. 10.1080/16549716.2019.1633725 31328678 PMC6713952

[22] Atre S. Tuberculosis burden in India's private sector. Lancet Infect Dis. (2016) 16:1328–9. 10.1016/S1473-3099(16)30470-4 27998589

[23] Government of India. Guidance Document on Partnerships. Revised National Tuberculosis Control Programme. Ministry of Health and Family Welfare. New Delhi (2019). Retrieved from https://tbcindia.gov.in/showfile.php?lid=3456 (accessed December 12, 2020).

