# Supplementary material for: Rising Catastrophic Expenditure on Households Due to Tuberculosis: Is India Moving Away From the END-TB Goal?
Source: Front Public Health. 2021 Feb 15;9:614466. doi: 10.3389/fpubh.2021.614466 (PMC7917129; doi:10.3389/fpubh.2021.614466)
Supplement: Supplementary file 1 [file Data_Sheet_1.docx]

**Supplementary Table:**

Table S 1: Quintile wise access to medical care across 52^nd^, 60^th^, 71^st^ and 75^th^ Rounds

|  | **52^nd^ Round** | | | | | | **60^th^ Round** | | | | | | **71^st^ Round** | | | | | | **75^th^ Round** | | | | | |
| --- | --- | --- | --- | --- | --- | --- | --- | --- | --- | --- | --- | --- | --- | --- | --- | --- | --- | --- | --- | --- | --- | --- | --- | --- |
| Quintile# | Public OPD (%) | Private OPD  (%) | Total*  OPD  (%) | Public IPD  (%) | Private IPD  (%) | Total*  IPD  (%) | Public OPD (%) | Private OPD  (%) | Total*  OPD  (%) | Public IPD  (%) | Private IPD  (%) | Total*  IPD  (%) | Public OPD (%) | Private OPD  (%) | Total*  OPD  (%) | Public IPD  (%) | Private IPD  (%) | Total*  IPD  (%) | Public OPD (%) | Private OPD  (%) | Total OPD (%) | Public IPD  (%) | Private IPD  (%) | Total IPD  (%) |
| First quintile | 27 (50%) | 23 (42%) | 54 (14%) | 146 (75%) | 46 (24%) | 194 (26%) | 33 (72%) | 13 (28%) | 46 (36%) | 172 (63%) | 101 (37%) | 273 (30%) | 43 (60%) | 29 (40%) | 72 (25%) | 96 (67%) | 48 (33%) | 144 (28%) | 46  (68%) | 22  (32%) | 68  (26%) | 86  (71%) | 35  (29%) | 121  (20%) |
| Second quintile | 42 (50%) | 37 (44%) | 84 (22%) | 128 (73%) | 43 (24%) | 176 (23%) | 13 (54%) | 11 (46%) | 24 (19%) | 149 (64%) | 84 (36%) | 233 (26%) | 32 (49%) | 33 (51%) | 65 (23%) | 73 (54%) | 61 (45%) | 134 (26%) | 34 (71%) | 14  (29%) | 48  (19%) | 86  (70%) | 37  (30%) | 123  (20%) |
| Third quintile | 40 (49%) | 34 (42%) | 81 (22%0 | 91 (61%) | 57 (38%) | 150 (20%) | 14 (48%) | 15 (52%) | 29 (23%) | 101 (57%) | 76 (43%) | 177 (20%) | 34 (60%) | 22 (39%) | 56 (20%) | 53 (63%) | 31 (37%) | 84 (16%) | 32  (59%) | 22  (41%) | 54  (21%) | 84  (67%) | 41  (33%) | 125  (20%) |
| Fourth quintile | 30 (38%) | 46 (58%) | 79 (21%) | 90 (63%) | 48 (34%) | 142 (19%) | 10 (56%) | 8 (44%) | 18 (14%) | 88 (64%) | 50 (36%) | 138 (15%) | 22 (47%) | 25 (53%) | 47 (16%) | 45 (58%) | 33 (42%) | 78 (15%) | 35  (78%) | 10  (22%) | 45  (17%) | 88  (70%) | 37  (30%) | 125  (20%) |
| Fifth quintile | 33 (43%) | 40 (52%) | 77 (21%) | 45 (48%) | 47 (50%) | 93 (12%) | 5 (50%) | 5 (50%) | 10 (8%) | 40 (50%) | 40 (50%) | 80 (9%) | 28 (59%) | 19 (40%) | 47 (16%) | 42 (53%) | 37 (47%) | 79 (15%) | 21  (50%) | 21  (50%) | 42  (16%) | 68  (59%) | 48  (41%) | 116  (19%) |
| Overall | 172 (46%) | 180 (48%) | 375 (100%) | 500 (66%) | 241 (32%) | 755 (100%) | 75 (59%) | 52 (41%) | 127 (100%) | 550 (61%) | 351 (39%) | 901 (100%) | 159 (55%) | 128 (45%) | 287 (100%) | 309 (60%) | 210 (40%) | 519 (100%) | 168 (61) | 89 (32%) | 257  (100%) | 412  (67%) | 198  (33%) | 610  (100%) |

* Column percentage, # Row percentage The proportion of mean expenditure for medical care across NSSO rounds.

**Table S 2 Comparison of medical related cost of each episode of outpatient care due to tuberculosis across wealth quintiles in four national surveys of India from 1995-1996 to 2017-2018**

|  | Overall expenditure | | | | Public sector | | | | Private sector | | | |
| --- | --- | --- | --- | --- | --- | --- | --- | --- | --- | --- | --- | --- |
|  | 52^nd^ round | 60^th^ round | 71^st^ round | 75^th^ round | 52^nd^ round | 60^th^ round | 71^st^ round | 75^th^ round | 52^nd^ round | 60^th^ round | 71^st^ round | 75^th^ round |
|  | N=375 | N=127 | N=287 | N=275 | N=172 | N=75 | N=159 | N=168 | N=180 | N=52 | N=128 | N=89 |
| First quintile | 0(0-15) | 6(0-15) | 3(3-5) | 13(8-51) | 0(0-7) | 3(0-12) | 3(0-4) | 1(1-14) | 20(3-39) | 6(2-19) | 4(3-9) | 13(13-51) |
| Second quintile | 10(0-16) | 6(0-9) | 9(3-14) | 25(17-123) | 10(3-20) | 0(0-4) | 3(3-10) | 123(123-123) | 13(13-16) | 9(2-12) | 9(3-14) | 17(17-25) |
| Third quintile | 1(0-16) | 12(3-18) | 16(7-16) | 7(2-9) | 0.7(0-5) | 0(0-9) | 8(3-38) | 7(0-8) | 19(10-52) | 12(2-21) | 16(7-16) | 5(2-9) |
| Fourth quintile | 7(0-13) | 58(36-58) | 5(0-24) | 0(0-8) | 12(7-21) | 68(5-68) | 1(0-3) | 0(0-0) | 13(9-23) | 58(2-58) | 24(13-24) | 8(8-25) |
| Fifth quintile | 12(0-33) | 39(0-58) | 1(1-6) | 43(5-370) | 8(0-10) | 43(0-194) | 1(1-1) | 0(0-2) | 49(16-124) | 39(2-39) | 9(6-26) | 370(35-370) |

Note: 52^nd^ Round was conducted b/w 1995-1996 and CPI unit used is 4.23 to equate to 2017 value. 60^th^ Round was conducted b/w 2004-2005 and CPI unit used is 2.52 to equate to 2017 value. 71^st^ Round was conducted b/w 2014-2015 and CPI unit used is 1.139 to equate to 2017. The annual average of USD exchange rate for 2017 is 65.12 to INR is used across the rounds. Medical related cost includes – Consultation Fee, Cost of medicines, diagnostic tests, attendant charges, physiotherapy, personal medical appliances, blood, oxygen, etc The figures are in median expenditure and interquartile range (IQR) is in parentheses.

**Table S 3 Comparison of medical related cost of each episode of hospitalization due to tuberculosis across wealth quintiles in four national surveys of India from 1995-1996 to 2017-2018**

|  | Overall expenditure | | | | Public sector | | | | Private sector | | | |
| --- | --- | --- | --- | --- | --- | --- | --- | --- | --- | --- | --- | --- |
|  | 52^nd^ round | 60^th^ round | 71^st^ round | 75^th^ round | 52^nd^ round | 60^th^ round | 71^st^ round | 75^th^ round | 52^nd^ round | 60^th^ round | 71^st^ round | 75^th^ round |
|  | N=755 | N=901 | N=520 | N=610 | N=500 | N=550 | N=310 | N=610 | N=241 | N=351 | N=210 | N=610 |
| First quintile | 55(11-101) | 68(29-167) | 70(29-152) | 63(10-279) | 39(7-98) | 46(12-116) | 54(10-96) | 38(5-107) | 78(33-164) | 110(68-261) | 157(73-612) | 264(98-668) |
| Second quintile | 78(33-176) | 97(37-232) | 70(18-166) | 60(5-220) | 49(20-98) | 58(12-120) | 18(18-98) | 23(2-73) | 228(91-390) | 246(97-341) | 163(70-255) | 220(111-726) |
| Third quintile | 130(20-260) | 86(24-214) | 87(30-259) | 138(30-226) | 52(0-195) | 38(6-112) | 35(14-157) | 31(4-92) | 234(143-325) | 155(97-283) | 145(66-315) | 226(226-362) |
| Fourth quintile | 98(42-260) | 213(53-628) | 101(5-339) | 61(23-184) | 98(39-195) | 53(17-165) | 8(4-70) | 38(13-74) | 104(33-325) | 542(244-1937) | 241(101-640) | 240(92-384) |
| Fifth quintile | 325(163-975) | 107(39-329) | 285(68-308) | 82(23-232) | 390(163-975) | 107(39-535) | 308(68-308) | 23(9-86) | 293(182-975) | 124(52-294) | 285(87-297) | 230(107-537) |

Note: 52^nd^ Round was conducted b/w 1995-1996 and CPI unit used is 4.23 to equate to 2017 value. 60^th^ Round was conducted b/w 2004-2005 and CPI unit used is 2.52 to equate to 2017 value. 71st Round was conducted b/w 2014-2015 and CPI unit used is 1.139 to equate to 2017. The annual average of USD exchange rate for 2017 is 65.12 to INR is used across the rounds. Medical related cost includes – Consultation Fee, Cost of medicines, diagnostic tests, attendant charges, physiotherapy, personal medical appliances, blood, oxygen, etc The figures are in median expenditure and interquartile range (IQR) is in parentheses.

**Table S 4 Proportion of expenditure on medicines to total hospital expenditure due to hospitalization for tuberculosis**

**across wealth quintiles in three national surveys of India from 1995-1996 to 2017-2018**

|  | 60^th^ Round | | | 71^st^ Round | | | 75^th^ Round | | |
| --- | --- | --- | --- | --- | --- | --- | --- | --- | --- |
| Quintile | overall | Public | Private | overall | Public | Private | overall | Public | Private |
| First quintile | 19% | 21% | 18% | 30% | 41% | 24% | 29% | 26% | 35% |
| Second quintile | 11% | 15% | 8% | 36% | 48% | 27% | 27% | 38% | 32% |
| Third quintile | 18% | 25% | 11% | 35% | 41% | 35% | 34% | 23% | 23% |
| Fourth quintile | 7% | 16% | 5% | 31% | 17% | 35% | 40% | 62% | 38% |
| Fifth quintile | 7% | 9% | 5% | 27% | 31% | 24% | 35% | 32% | 18% |
| overall | **11**% | 16% | 8% | **31%** | 37% | 28% | **29%** | 45% | 36% |
